# Higher sensitive selective spectrofluorometric determination of ritonavir in the presence of nirmatrelvir: application to new FDA approved co-packaged COVID-19 pharmaceutical dosage and spiked human plasma

**DOI:** 10.1186/s13065-023-01030-0

**Published:** 2023-09-21

**Authors:** Mohamed S. Imam, Ahmed H. Abdelazim, Sherif Ramzy, Ahmed A. Almrasy, Mohammed Gamal, Afnan S. Batubara

**Affiliations:** 1 Pharmacy Practice Department, College of Pharmacy, Shaqra University, Shaqra, 11961 Saudi Arabia; 2 Clinical Pharmacy Department, National Cancer Institute, Cairo University, Fom El Khalig Square, Kasr Al-Aini Street, Cairo, 11796 Egypt; 3 Pharmaceutical Analytical Chemistry Department, Faculty of Pharmacy, Al-Azhar University, Cairo, 11751 Egypt; 4 Pharmaceutical Analytical Chemistry Department, Faculty of Pharmacy, Beni-Suef University, Beni-Suef, 62514 Egypt; 5 Department of Pharmaceutical Chemistry, College of Pharmacy, Umm Al-Qura University, Makkah, 21955 Saudi Arabia; 6 Nasr City, Cairo, 11751 Egypt

**Keywords:** Ritonavir, Fluorescent nature, Nano level, Spiked plasma

## Abstract

**Background:**

Ritonavir was recently combined with nirmatrelvir in a new approved co-packaged medication form for the treatment of COVID-19. Quantitative analysis based on fluorescence spectroscopy measurement was extensively used for sensitive determination of compounds exhibited unique fluorescence features.

**Objective:**

The main objective of this work was to develop higher sensitive cost effective spectrofluorometric method for selective determination of ritonavir in the presence of nirmatrelvir in pure form, pharmaceutical tablet as well as in spiked human plasma.

**Methods:**

Ritonavir was found to exhibit unique native emission fluorescence at 404 nm when excited at 326 nm. On the other hand, nirmatrelvir had no emission bands when excited at 326 nm. This feature allowed selective determination of ritonavir without any interference from nirmatrelvir. The variables affecting fluorescence intensity of ritonavir were optimized in terms of sensitivity parameters and principles of green analytical chemistry. Ethanol was used a green solvent which provided efficient fluorescence intensity of the cited drug.

**Results:**

The method was validated in accordance with the ICH Q2 (R1) standards in terms of linearity, limit of detection (LOD), limit of quantification (LOQ), accuracy, precision and specificity. The described method was successfully applied for ritonavir assay over the concentration range of 2.0–20.0 ng/mL.

**Conclusion:**

Ritonavir determination in the spiked human plasma was successfully done with satisfactory accepted results.

## Introduction

Fluorescence spectroscopy provides a more sensitive analytical tool for the determination of many compounds, especially those that have a unique fluorescent nature. The high sensitivity of fluorescence spectroscopy is the main advantage, as it allows the determination of compounds down to the Nano gram range [1–3]. In addition, the fluorescence spectroscopy instrument consumes less than 0.1 kWh of energy per sample and produces little waste, which draws attention to the principles of green chemistry [4].

**Fig. 1:**
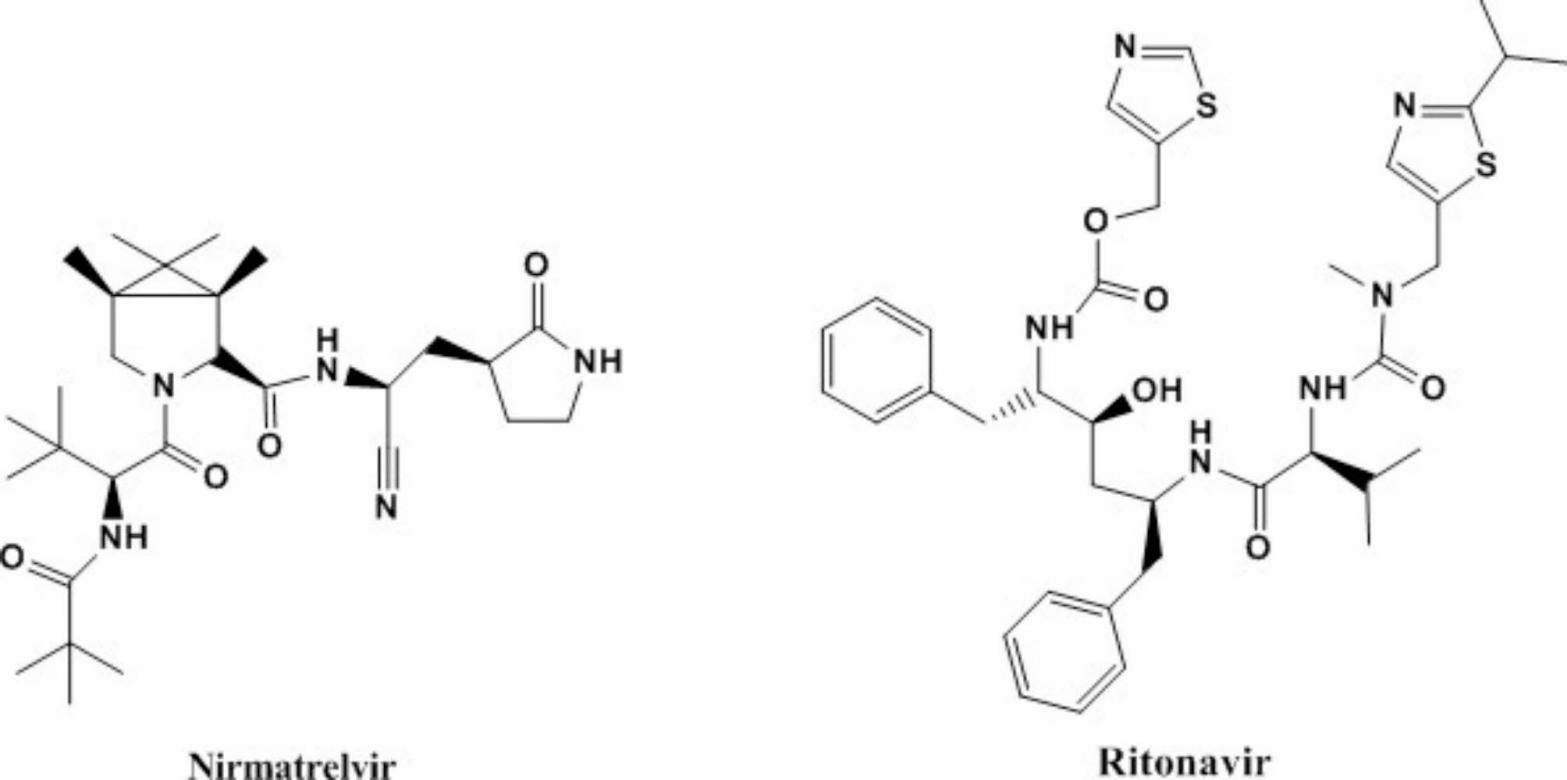
Structural formula of nirmatrelvir and ritonavir

Ritonavir plus nirmatrelvir, Fig. 1, is a new FDA co-packaged drug developed for the treatment of COVID − 19. Ritonavir is a protease inhibitor and CYP3A inhibitor of human immunodeficiency virus type 1, and nirmatrelvir is a peptidomimetic inhibitor of the major protease of severe acute respiratory syndrome coronavirus 2. Ritonavir plus nirmatrelvir received the first conditional marketing authorization in the United Kingdom in December 2021 for the treatment of COVID − 19 in adults who do not require supplemental oxygen and are at high risk for progression to severe COVID − 19. Nirmatrelvir plus ritonavir was also approved in Europe and the United States for COVID-19 [5–8].

Previous reports have described various analytical methods for the determination of ritonavir in different matrices. HPLC method has been developed for the determination of ritonavir in human plasma [9] and pharmaceutical dosage forms [10]. Although the HPLC method was widely used for the determination of active pharmaceutical ingredients, it had several disadvantages, such as the high time required, the recommended sample pretreatment, and the possibility of determining active ingredients in the microgram range. Moreover, the direct UV spectrophotometric method [11] and mathematical manipulation of spectral bands including first-order derivative and area under the curve [12] were used for the determination of ritonavir in the pharmaceutical dosage form in the microgram range. Despite the simple approach and affordability of the spectrophotometric methods, they could not provide a feasible determination of ritonavir in the biological samples.

The main objective of this work was to develop a more sensitive spectrofluorometric method for the selective determination of ritonavir in the presence of nirmatrelvir in pure form, in pharmaceutical tablets, and in spiked human plasma. The method was based on the unique fluorescence properties of ritonavir, the compound of interest, and the absence of any fluorescence properties of nirmatrelvir, another compound packaged with ritonavir in a new pharmaceutical dosage form. This feature allowed direct selective determination of ritonavir without any interference from nirmatrelvir. The proposed method was efficiently used for the determination of ritonavir up to the Nano- gram range in the plasma matrix and pharmaceutical dosage forms. Moreover, the proposed method showed superiority over the previously reported spectrophotometric method in terms of sensitivity and quantification limits.

## Experimental

### Materials and chemicals

- Pure reference standard of nirmatrelvir (99.36%) and ritonavir (99.62%) were kindly supplied by Pfizer, Inc, Egypt. The purity was checked by applying the reported method [13].
- Paxlovid tablets, pink oval nirmatrelvir film-coated tablet (150 mg) co-packaged with white ritonavir film-coated tablet (100 mg), (B. NO: A1324, manufactured by Pfizer Company), were kindly supplied by Pfizer, Inc., Egypt. The recommended dose is two tablets of nirmatrelvir plus one tablet of ritonavir.
- Acetonitrile, chloroform, ethanol and methanol (Sigma-Aldrich, Germany).
- Tween 80, Sodium dodecyl sulphate “SDS”, El-Nasr Company, Egypt. Cetyl trimethyl ammonium bromide “CTAB” (Win lab, UK). β-cyclodextrin “β-CD”, Sigma-Aldrich, Germany.
- Hydrochloric acid and Sodium hydroxide, El-Nasr Company, Egypt. 0.1 N aqueous solutions were prepared.
- Boric acid, glacial acetic acid, potassium chloride acid and sodium acetate trihydrate, El-Nasr Company, Egypt.
- Acetate buffer solutions in the pH range 4 to 6 and alkaline borate buffer in the pH range 8 to 10.
- Drug-free human plasma of healthy volunteers was supplied by Blood Bank, Al-Azhar University Hospital, Damietta, Egypt.

### Instrumentation

Spectrofluorometric apparatus, Jasco FP-6200, Tokyo, Japan.

### Standard solutions

Prepared stock solutions of ritonavir and nirmatrelvir [100 µg/mL] were prepared by dissolving 10 mg of ritonavir or nirmatrelvir in 50 mL of ethanol and adding ethanol to a 100-mL volumetric flask. Definitive working solutions were prepared by dilution with ethanol.

### Procedures

#### Calibration graph construction

Specific volumes of ritonavir stock solutions were added to a series of 10-mL volumetric flasks to produce a series of solutions with accurate concentrations ranging from 20.0to 20.0 ng/mL. The solutions were excited at 326 nm and the intensity of fluorescence emission was measured at 404 nm. The values of the evaluated emission intensity were plotted against the corresponding ritonavir concentrations and the regression equations were derived.

#### Laboratory prepared mixture analysis

Laboratory mixtures of ritonavir and nirmatrelvir were prepared in various ratios, assuming that the recommended dose consisted of two tablets of nirmatrelvir and one tablet of ritonavir, corresponding to a nirmatrelvir to ritonavir ratio of 3:1. The mixtures were tested using the procedures described, and the drug concentration was determined using the regression equation. In general, all procedures described were performed in accordance with the relevant guidelines.

#### Procedures for tablets

Two units of ritonavir tablets were scratched, weighed, and finely powdered. In a 100-mL volumetric flask, the drug powder equivalent to one tablet was dissolved in 30 mL ethanol and sonicated for 20 min. The volume was made up to 100 mL with ethanol to obtain a solution containing 1 mg/mL ritonavir. Finally, five concentrations of ritonavir were prepared and evaluated as previously described.

#### Procedure for spiked human plasma

Various spiked human plasma samples were prepared by placing aliquots of different concentrations of ritonavir together with 1 mL of human plasma and 3 mL of acetonitrile in a series of 10-mL centrifugation tubes. The tubes were shaken in a vortex mixer for 1 min and centrifuged for 30 min. The resulting supernatant was evaporated to dryness, and the residues were dissolved in a fixed volume of ethanol. In 10-mL volumetric flasks, 3 mL of acetate buffer pH 4 was added and made up with ethanol. Samples were evaluated as previously described.

## Results and discussion

Many efforts have been made to improve the sensitivity of quantitative analysis of drugs. Spectrophotometry and spectrofluorometry are considered the most commonly used analytical techniques in pharmaceutical analysis because they are simple and inexpensive [14–24]. In addition, spectrofluorometry is a preferred technique for compounds with unique native fluorescent features in terms of higher sensitivity metrics and lower detection limits [2, 3].

In this work, a spectrofluorometric method for the efficient determination of ritonavir in the presence of nirmatrelvir in pure form, in pharmaceutical tablets, and in spiked human plasma was developed, adopted, and applied. The described method was based on the fluorescence properties of ritonavir, whereas the coexisting drug nirmatrelvir showed no fluorescence behavior. This property allowed selective determination of ritonavir without any interference from nirmatrelvir.

### Fluorescence characteristics

**Fig. 2:**
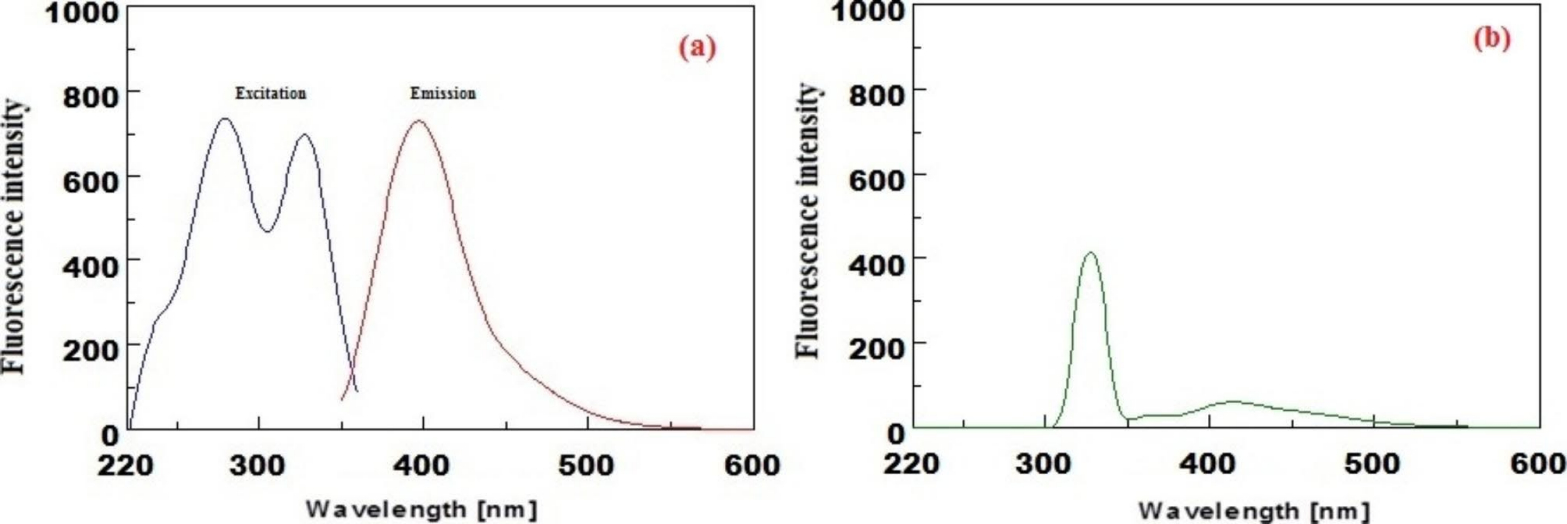
Excitation and emission spectra of 15 ng/mL ritonavir (**a**) and 45 ng/mL nirmatrelvir (**b**)

Ritonavir showed a unique native emission spectrum at 404 nm when excited at 326 nm (see Fig. 2). (a) Nirmatrelvir, on the other hand, had no emission bands when excited at 326 nm (see Fig. 2). (b) The obtained emission bands of ritonavir allowed a more sensitive and selective approach for its determination in the pharmaceutical dosage form as well as in spiked human plasma without any interference from nirmatrelvir.

### Factors affecting the fluorescence properties

#### Diluting solvent effect

**Fig. 3:**
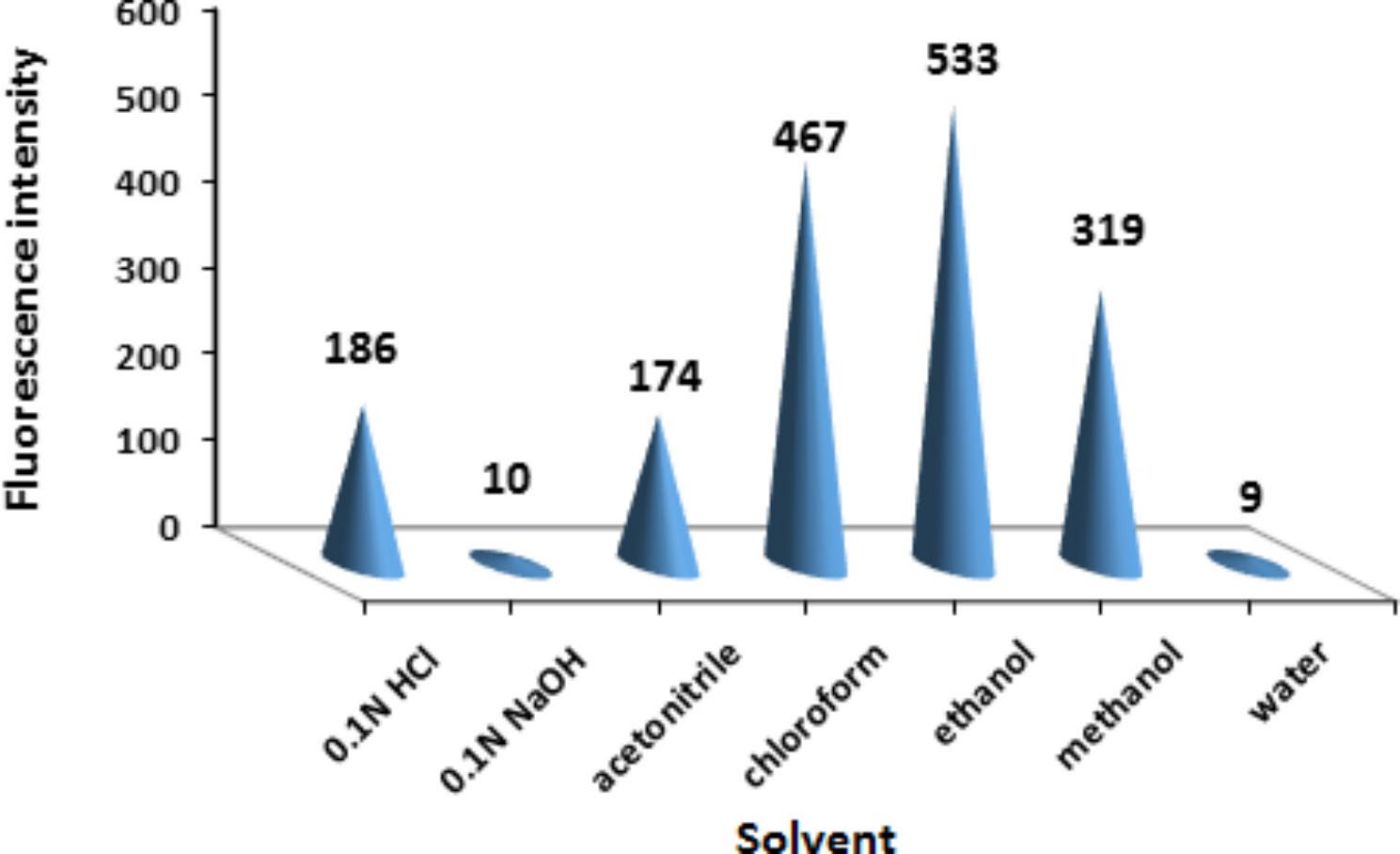
Effect of different diluting solvents on fluorescence intensity of 15 ng/mL ritonavir

The effect of different diluents such as water, acetonitrile, methanol, ethanol, chloroform, 0.1 N HCl, and 0.1 N NaOH on the fluorescence efficiency of ritonavir was tested. Complete quenching of fluorescence was observed when 0.1 N NaOH was used. Water did not result in quantitative feasibility of the cited drug. On the other hand, chloroform and ethanol were found to be the best dilution solvents as they exhibited higher fluorescence intensity with lower blank values. Since chloroform had undesirable environmental and health effects, ethanol was selected due to its environmental green superiority [25]. The effect of the different diluents is shown in Fig. 3.

#### Surfactant effect

**Fig. 4:**
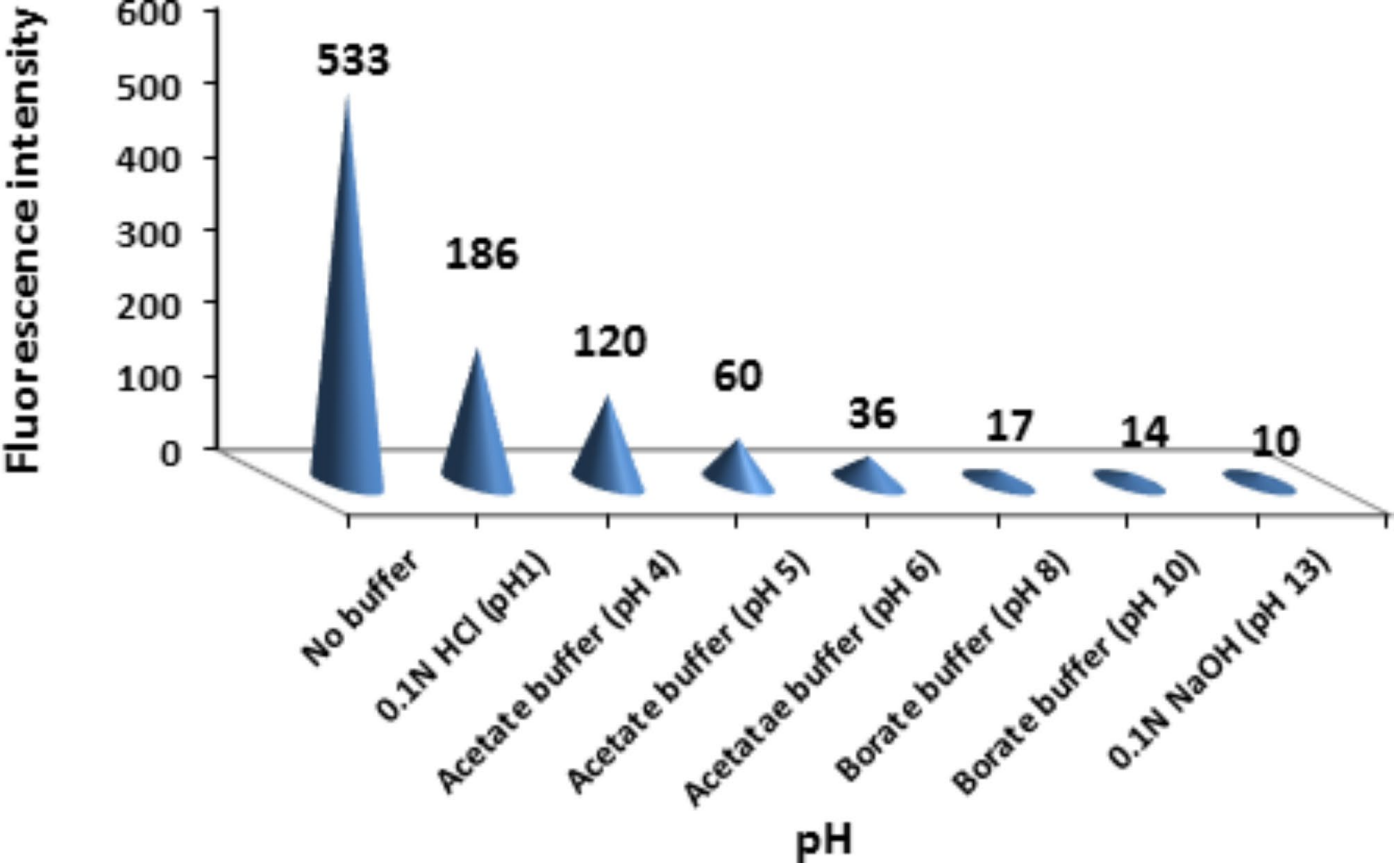
Effect of different buffer types on fluorescence intensity of 15 ng/mL ritonavir

The effect of adding surfactants including SDS, CTAB, Tween 80 and β-CD on the ritonavir fluorescence efficiency were tested. Adding of these surfactants resulted in fluorescence quenching of ritonavir. So there was no need for adding any surfactant, Fig. 4.

#### pH effect

**Fig. 5:**
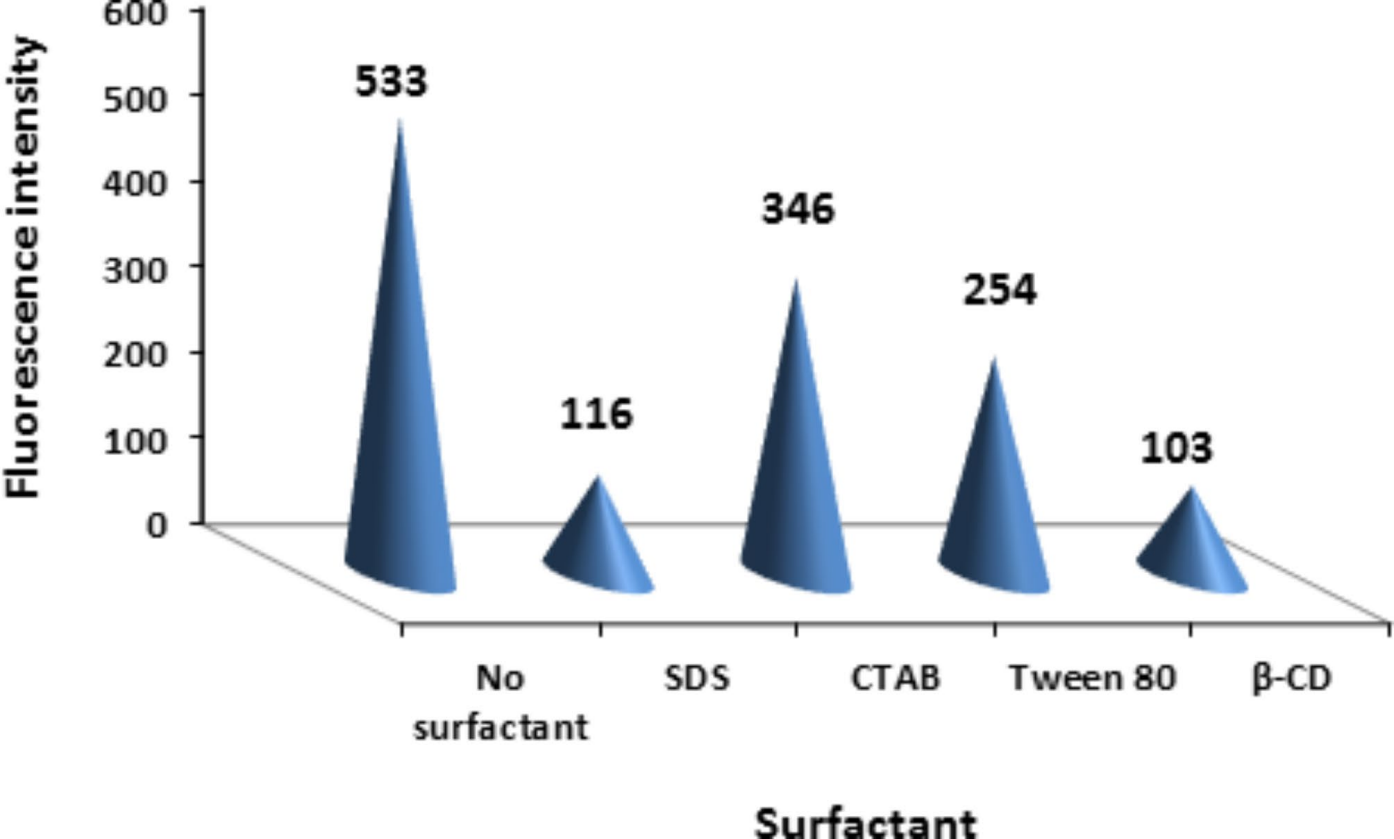
Effect of different surfactant types on fluorescence intensity of 15 ng/mL ritonavir

Various types of buffer at several pH values were tested. The fluorescence intensity of ritonavir was decreased by adding any type of buffer solution. So there was no need for adding any surfactant, Fig. 5.

#### Method validation

The method was validated in accordance to ICH guidelines [26]. The linearity was tested by analyzing five concentration sets of ritonavir. The fluorescence intensity was plotted against drug concentration and the linearity was obtained over a concentration range of 2.0–20.0 ng/mL. The regression parameters were assessed and the obtained results revealed acceptable values as listed in Table 1. Limit of quantitation [LOQ] was defined as the lowest concentration of compound of interest which can be accurately analyzed. While the limit of detection [LOD] was defined as the lowest concentration of compound of interest which can be detected. Values were calculated and listed in Table 1. Accuracy, expressed as mean percent recovery [%R], was determined by applying the proposed procedure for triplicate determinations of [5, 10,15 ng/mL] ritonavir. The %R was calculated and presented in Table 2. Precision, as percent relative standard deviation [%RSD], was determined by determining [5, 10,15 ng/mL] ritonavir. For repeatability, it was performed within one day and for mean precision, it was performed on three consecutive days. The lower values of %RSD prove the higher precision of the described method, as shown in Table 1.

**Table 1:** Regression and validation data for determination of ritonavir by the proposed method

Parameters	Obtained data
Emission wavelength (nm)	404
Excitation wavelength (nm)	326
Slope	40.41
Intercept	28.25
Coefficient of determination(r^2^)	0.9994
Accuracy (mean %R) ^*^	99.24
Repeatability (RSD)^*^	0.854
Intermediate precision (RSD)^*^	0.925
LOD (ng/mL)	0.146
LOQ (ng/mL)	0.444
Range (ng/mL)	2.0–20.0

^*^ Values corresponding to three determinations of three concentrations levels

**Table 2:** Determination of ritonavir in laboratory prepared mixtures with nirmatrelvir using the described method

Ritonavir (ng/mL)	Nirmatrelvir (ng/mL)	Ritonavir found (ng/mL)	Ritonavir [% Recovery]
3.00	9.00	2.97	99.00
4.00	12.00	4.03	100.75
5.00	15.00	5.05	101.00
6.00	18.00	5.92	98.66
Mean ± %RSD			99.85 ± 1.859

#### Selective determination of ritonavir in a laboratory prepared mixture with nirmatrelvir

The described method was selectively applied to the determination of ritonavir in a laboratory prepared mixture with nirmatrelvir, assuming that the recommended dose consisted of two tablets of nirmatrelvir and one tablet of ritonavir, corresponding to a nirmatrelvir to ritonavir ratio of 3: 1. The results were in good agreement with the label claim as listed in Table 2.

#### Method application for ritonavir determination in the pharmaceutical dosage form and spiked human plasma

The method described was applied to determine ritonavir in the pharmaceutical dosage form, Table 3. The results were in good agreement with statistical accepted results in comparison to the reported spectrophotometric method [11]. Furthermore, the method was applied for the determination of ritonavir in spiked human plasma regarding to the high sensitivity detection limits which allowed the determination of ritonavir in the plasma matrix. The data revealed selective determination of ritonavir without interference of endogenous components of the plasma matrix as listed in Table 4.

**Table 3:** Application of the described method for determination of ritonavir in the pharmaceutical preparation with statistical assessment of the obtained results with the others obtained by the reported one

Parameter	Described Method	Reported method [11]
Mean ^a^	100.13	100.32
SD ^a^	1.063	1.398
Variance ^a^	1.1235	1.958
*t-*test	0.248 (2.306)^b^	------
*F-*value	1.718 (6.388)^b^	

^a^ Mean recovery, % of five concentration sets

^b^ The values in the parenthesis are the corresponding theoretical values of *t* and *F* at (*P* = 0.05)

**Table 4:** Determination of ritonavir, in presences of nirmatrelvir, in spiked human plasma matrix

Ritonavir (ng/mL)	Nirmatrelvir (ng/mL)	Ritonavir found (ng/mL)	Ritonavir [% Recovery]
3.00	9.00	2.82	94.00
4.00	12.00	3.85	96.25
5.00	15.00	4.86	97.20
6.00	18.00	6.14	102.33
Mean ± %RSD			97.45 ± 2.25

#### Comparative evaluation of the described method with previously reported spectrophotometric method

In general, the spectrofluorometric procedures described represented the first analytical method to selectively determine ritonavir in the presence of nirmatrelvir in its recently FDA-approved co-packaged form and spiked human plasma. The current results show that the nano- level determination of ritonavir has higher sensitivity compared to previously reported analytical methods. In particular, in terms of sensitivity, detection limits and quantification, the proposed method was compared with the previously used spectrophotometric method [11]. The proposed method allowed the determination of ritonavir in a concentration range of 2–20 ng/mL with a lower limit of detection up to 0.146 ng/mL and a limit of quantification up to 0.444 ng/mL, which confirmed its higher sensitivity compared to the spectrophotometric method (see Table 5). In addition, ethanol was chosen as the solvent in the described method compared to methanol, which was used as the solvent in the reported method. Ethanol is less toxic, has lower vapor pressure and evaporation, resulting in lower inhaled amount with lower disposal cost and high environmental friendliness [21, 27]. This advantage shows the environmental friendliness of the described method compared to previous methods.

**Table 5:** Comparative evaluation of the described method with previously reported spectrophotometric method

Parameters	Proposed method	Reported method [11]
Solvent	Ethanol	Methanol
Matrix	Pharmaceutical form and spiked plasma	Pharmaceutical form
Linearity range	2.0 − 20.0 ng/mL	10.0 − 20.0 µg/mL
LOD	0.146 ng/mL	1.100 µg/mL
LOQ	0.444 ng/mL	3.300 µg/mL

## Conclusion

In the described work, a more sensitive spectrofluorometric method for the selective determination of ritonavir in the presence of nirmatrelvir in pure form, in pharmaceutical tablets as well as in spiked human plasma was presented. The method was fully optimized, validated, and compared with the previously reported spectrophotometric method. In terms of sensitivity metrics, the described method was superior to published analytical methods in terms of Nano-level determination of ritonavir in various matrices.

## Data Availability

The datasets used and/or analyzed during the current study are available from the corresponding author on reasonable request.
